# Application of Metal-Organic Framework Nano-MIL-100(Fe) for Sustainable Release of Doxycycline and Tetracycline

**DOI:** 10.3390/nano7080215

**Published:** 2017-08-06

**Authors:** Seyed Dariush Taherzade, Janet Soleimannejad, Aliakbar Tarlani

**Affiliations:** 1 School of Chemistry, College of Science, University of Tehran, P.O. Box 14155-6455, Tehran, Iran; d.taherzade@ut.ac.ir; 2 Chemistry & Chemical Engineering Research Center of Iran (CCERCI), Pajoohesh Blvd., km 17, Karaj Hwy, P.O. Box 14968-13151, Tehran, Iran; tarlani@ccerci.ac.ir

**Keywords:** doxycycline monohydrate, drug delivery, nano-MIL-100, sustain release, Tetracycline hydrochloride

## Abstract

Nanostructures of MIL-100 were synthesized and used as a drug delivery platform for two members of the Tetracycline family. Doxycycline monohydrate (DOX) and Tetracycline hydrochloride (TC) were loaded separately on nano-MIL-100 (nanoparticles of drug@carrier were abbreviated as DOX@MIL-100 and TC@MIL-100). Characterizations were carried out using FT-IR, XRD, BET, DLS, and SEM. The FT-IR spectra revealed that the drugs were loaded into the framework of the carrier. The XRD patterns of DOX@MIL-100 and TC@MIL-100 indicated that no free DOX or TC were present. It could be concluded that the drugs are well dispersed into the pores of nano-MIL-100. The microporosity of the carrier was confirmed by BJH data. BET analysis showed a reduction in the free surface for both DOX@MIL-100 and TC@MIL-100. The release of TC and DOX was investigated, and it was revealed that MIL-100 mediated the drug solubility in water, which in turn resulted in a decrease in the release rate of TC (accelerating in DOX case) without lowering the total amount of released drug. After 48 h, 96 percent of the TC was sustain released, which is an unprecedented amount in comparison with other methods.

## 1. Introduction

Drug delivery is an approach in which a pharmaceutical compound is safely transported through the body to reach its target. Various techniques may be used, i.e., increasing the solubility of an insoluble drug, enhancing the duration of drug presence in the body, or optimizing the therapeutic effect [1]. A drug delivery system consists of two main parts: a carrier and a drug. Its favorable features are tuned by choosing the proper drug and carrier. Since different drugs require different treatments, numerous carriers have been incorporated. Until today, many carriers have been utilized, such as inorganic mesoporous silica, organic polymers, micelles, liposomes, and dendrimers [2,3,4,5,6]. However, the application of these conventional methods is limited due to their low cargo capacity and unchangeable release kinetics [7].

Metal-organic frameworks (MOFs) are able to overcome the limitations of organic and inorganic carriers [8]. The diverse methods that are used for the synthesis of MOFs provide the opportunity to design and control the chemical and physical properties of these materials [9,10]. The assembly of inorganic clusters and various organic linkers by strong bonds build MOFs. Their high porosities (φ up to 6 nm, pore volume up to 2.3 cm^3^ g^−1^) makes them great candidates for encapsulating pharmaceutical compounds and biomedical applications. The surface area could be further enhanced by reducing the dimensions to nanoscale [11,12]. Better drug interactions will be achieved as a consequence of engineering the structure, porosities, and hydrophobic/hydrophilic entities [13].

MIL-100 (MIL: Materials of Institut Lavoisier) was isolated as a polycrystalline powder from iron and benzene tricarboxylic acid by Horcajada et al. [14]. Large pores and a high surface area makes MIL-100 an ideal carrier for drug delivery purposes [15]. It has previously been used for encapsulating several drugs such as doxorubicin, ibuprofen, caffeine, and aspirin in high loading capacities compared to conventional methods [16,17].

The tetracycline class of compounds has different applications, like broad spectrum antibiotics that act against gram-positive and gram-negative bacteria. Although the bacterial resistance decreased, the demand for these antibiotics, they remained especially useful in the treatment of various bacterial infections such as chlamydia, acne, mycoplasma, periodontitis, and rickettsia. More recently, aspects of their “non-antibiotic applications,” such as on/off switching of the transcription of distinct genes, were also discovered [18]. Their structure is also very interesting due to several coordination sites that can bind to metals and establish non-covalent bonds [19]. There are several reports indicating that the pharmacological behavior of these compounds depends heavily on metal coordination sites. Ions like calcium and magnesium form complexes with tetracycline compounds and modify their antibiotic activities [20,21]. Considering these facts, it is very important to deliver these compounds to their target safely and sustain release them. Domingues and her co-workers used bioactive glass as a drug delivery system of tetracycline. The amount of released Tetracycline did not exceed 25% in 80 days in SBF solution [22]. In another approach, Aguzzi and his co-workers intercalated tetracycline into layered clay mineral material and used it as a platform for release. The release profile, in this case, showed a “burst effect” and their effort for slowing the release decreased the released amount to 60% [23].

In the current research, Tetracycline hydrochloride (TC) as a soluble drug and Doxycycline monohydrate (DOX) as a slightly soluble drug in water were encapsulated separately in nano-MIL-100, and their release was investigated. This MOF was chosen due to its compatibility (pore size of 1.7 nm and 1.2 nm) with the molecular size of tetracycline hydrochloride (1.10 nm) and doxycycline monohydrate (1.10 nm). Structural analysis revealed that these two antibiotics have same dimensions, and their difference is in some functional groups (Tables S1 and S2). TC has six hydrogen bond donor and nine hydrogen bond acceptor sites. Encapsulation of these drugs in nano-MIL-100 has two important advantages: (1) eliminating any chance of complexation by protecting them from ions and shielding the available coordination sites and (2) mediating their solubility in water which affects the release profile of both drugs.

## 2. Results and Discussion

**Figure 1:**
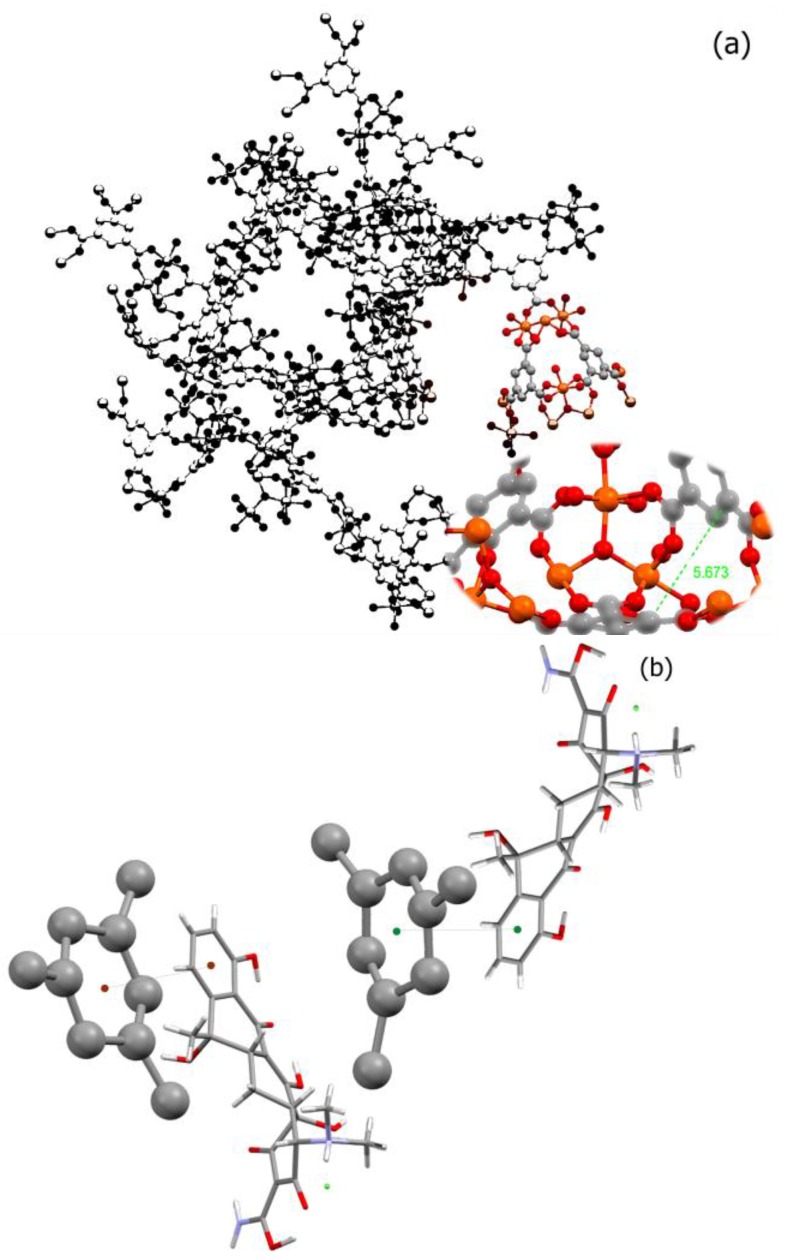
(**a**) The distance between two BTC rings in a pore of MIL-100; (**b**) the proposed π-π stacking between BTC rings (Ball and Stick) and TC rings (capped sticks).

Transition metal ions such as iron and copper are important components of biological processes. These processes have been implicated in many diseases including microbial infections, cancer, and neurodegenerative disorders. Considering the fact that many transition metals such as nickel, chromium, and cobalt are toxic and their high dosage in body would be harmful, choosing an appropriate metal for building a biological-friendly MOF could be a challenging issue. Among the suitable metals for building MOFs, we can name iron, zinc, calcium, magnesium, and manganese. Iron, however, has shown the capacity to form a safe and nontoxic cluster as a node for many MOFs including MIL-100. There are also several reports suggesting nano-MIL-100 (Fe) as a safe and stable capsule for several small molecule drugs. As in Figure 1a, benzene tricarboxylic ligands in MIL-100 have lost all of their hydrogens, and all acidic functional groups are attached to the metallic cluster [24]. Considering that the π-π interactions between aromatic rings act in the range of 3.5 Angstroms [25], and the distance between the two BTC rings in a pore of MIL-100 is about 5.763 Å, a non-covalent π-π stacking between BTC aromatic rings and aromatic rings of TC or DOX can be anticipated. Figure 1b illustrates a proposed orientation of TC in the MIL-100 pore which will favor a π-π stacking (distance of 2.67 Å) between its aromatic ring and BTCs. Therefore, it is perhaps this type of non-covalent interactions that holds a molecule of TC or DOX into the pore.

**Figure 2:**
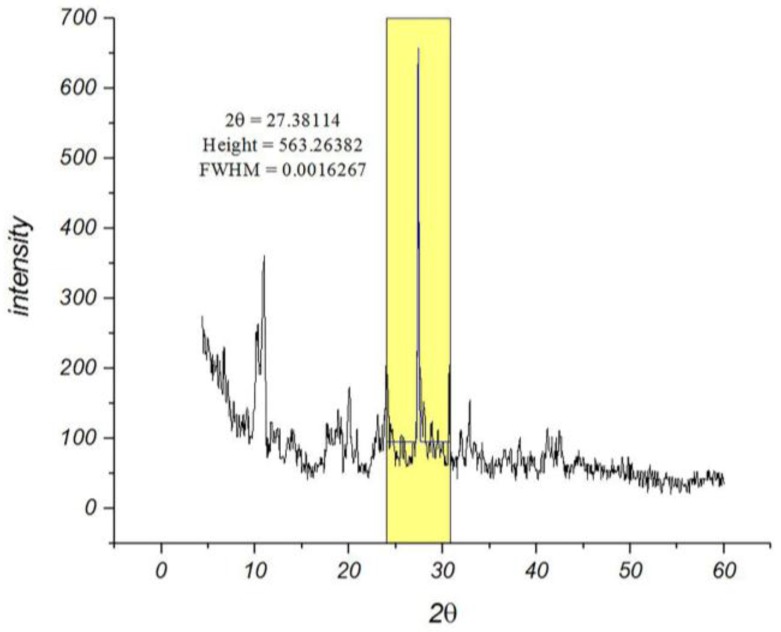
The XRD pattern of nano-MIL-100. The data for the main peak is provided.

**Figure 3:**
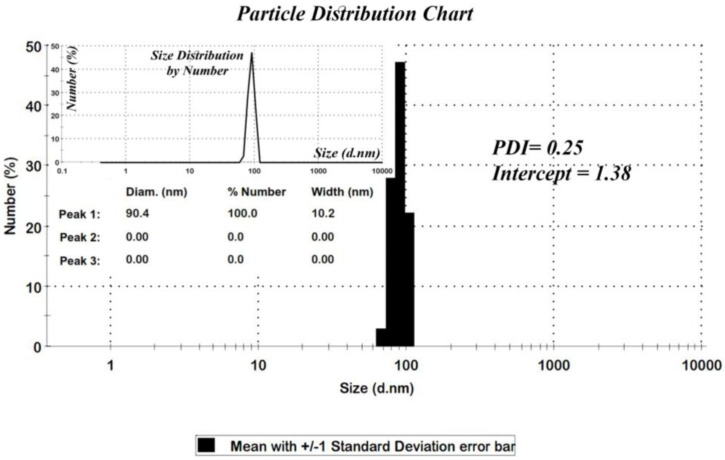
Size distribution histogram of nano-MIL-100.

Among the various methods that are used for determining the particle sizes, the X-ray diffraction pattern is a useful resource for crystalline materials [26]. Calculations on the XRD pattern of nano-MIL-100 (Figure 2) using Scherrer Equation confirm a nano-crystalline material with nano-sized domains (ES 1, Equation 1 in Supplementary materials). Calculations suggest that the mean size of the particles is approximately 88 nm. Further characterizations using Dynamic Light Scattering indicated that the mean size of particles is 90.4 nm. As seen in Figure 3, the distribution chart has a Gaussian distribution. As the polydispersity index (PDI) (0.25) suggests, there is a moderate polydispersity in the sample as a result of aggregation of nano particles which could explain the difference between theoretical calculations (Sherrer equation) and the results of scanning electron microscopy.

**Figure 4:**
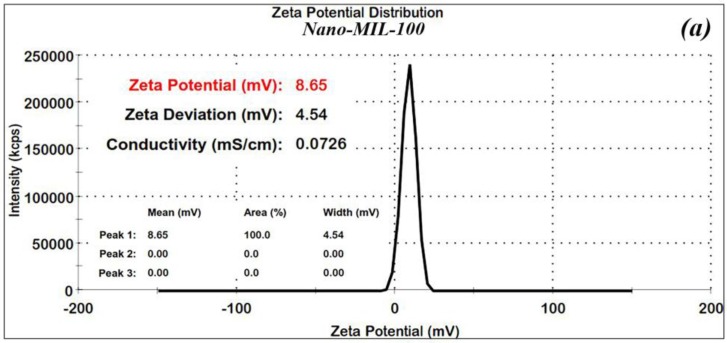

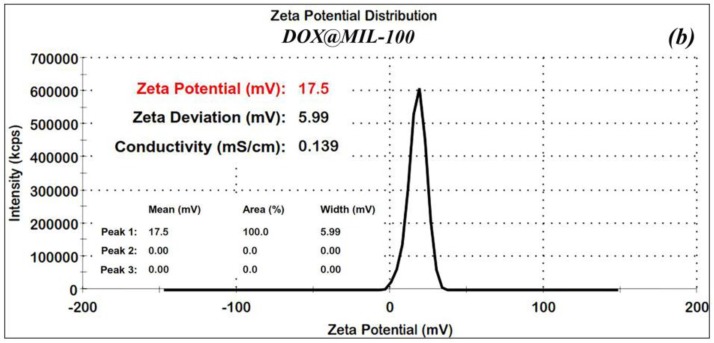
Zeta potential for nano-MIL-100 (**a**) and DOX@MIL-100 (**b**).

In order to confirm the stability of the nanoparticles, Zeta (ʓ) potential measurements were made for the nano-MIL-100 and DOX@MIL-100. The results, as shown in Figure 4a,b, reveal that Zeta potential values were found to be 8.6 and 17.5 mV, respectively. The ʓ potential in the range of ±10 to ±30 mV indicates incipient stability, which is expected for metal-organic frameworks, and the coagulation of nanoparticles in some regions is obvious in the SEM images and the PDI index. It is also noteworthy that in the current study, loading of DOX and TC into nanoparticles of MIL-100 causes an increase in the zeta potential, thus leading to a higher stability.

**Figure 5:**
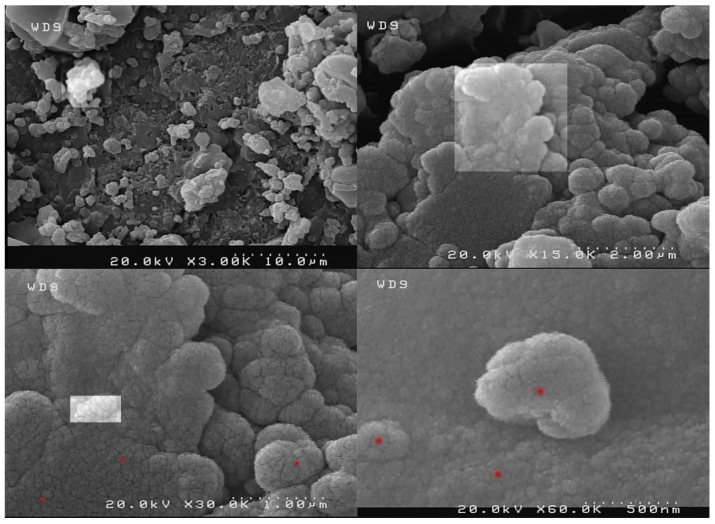
SEM image of nano-MIL-100. Extrapolation of SEM image showing magnification (from top-left to bottom-right). A number of nanoparticles are marked with red spots.

**Figure 6:**
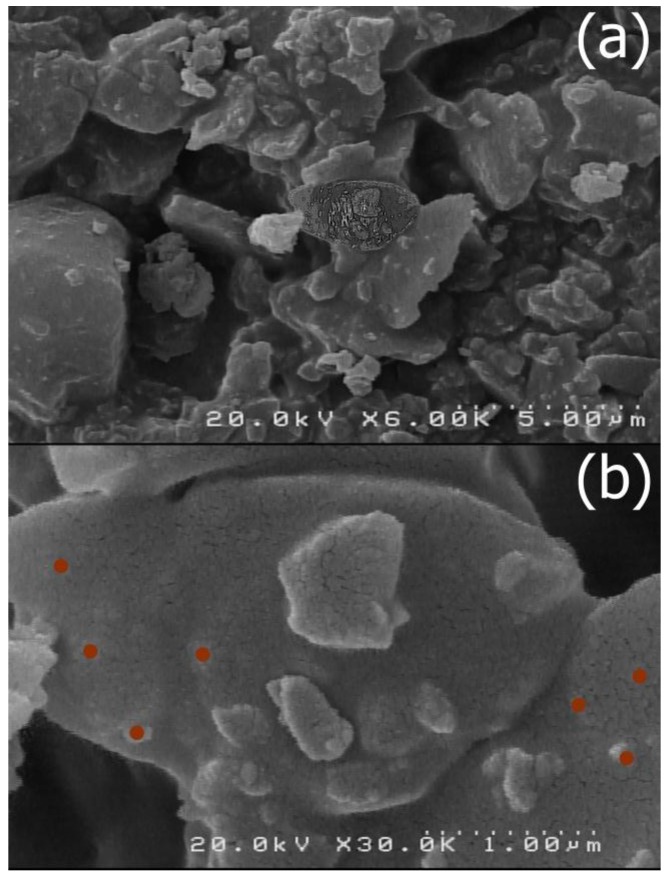
(**a**) SEM of TC@MIL-100; (**b**) Red spots indicate some of nanoparticles.

The size and morphology of the nano-MIL-100 compound was examined by scanning electron microscopy (SEM) and a kind of gel material could be seen in some regions probably based on nanoparticles (Figure 5). By comparing these results with the XRD pattern, it can be concluded that most of the particles are below 100 nm. Figure 6 represents the SEM Images of TC@MIL-100. The morphology of nano-MIL-100 remained intact, and some porosities that are obvious in Figure 5 are now filled with the molecules of the drug. By comparing the results of SEM and PDI, it is clear that the aggregation of particles in SEM images lead to a moderate polydispersity. As a result, it could be expected that some particles have different sizes.

**Figure 7:**
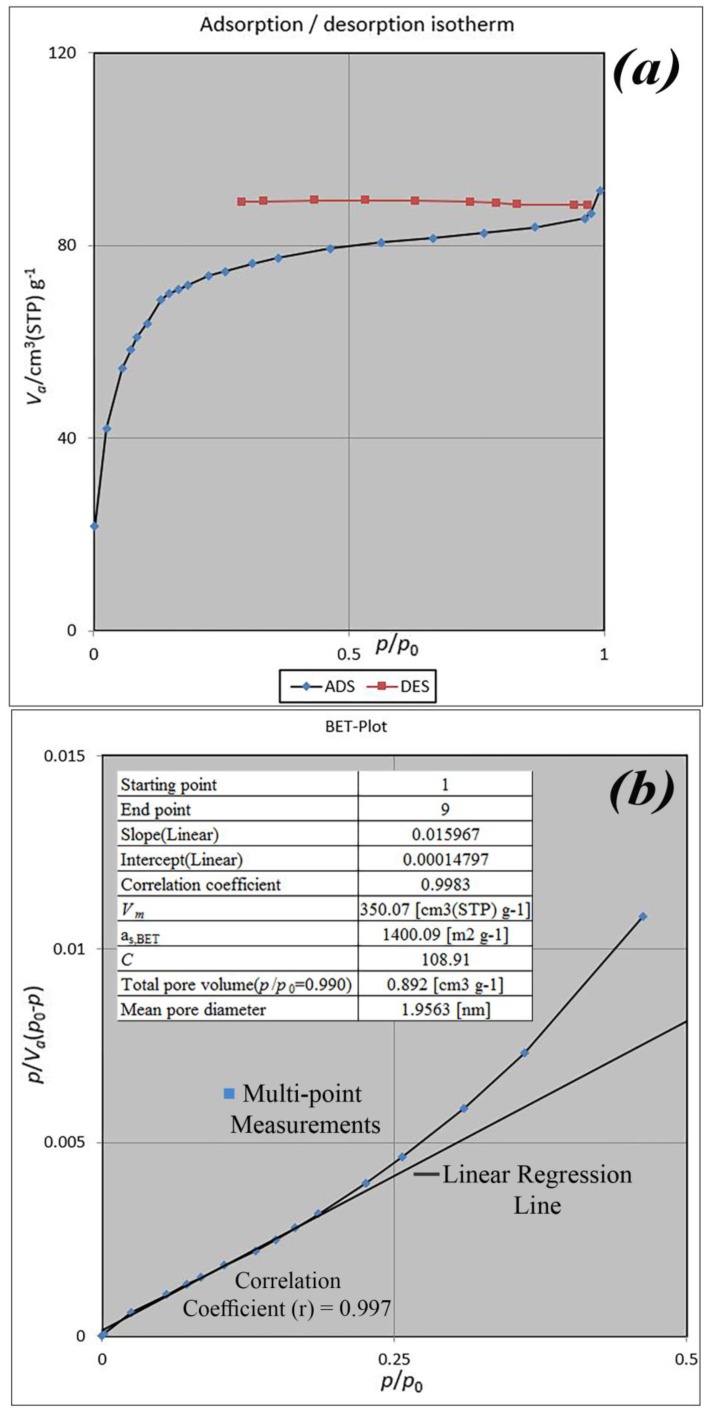

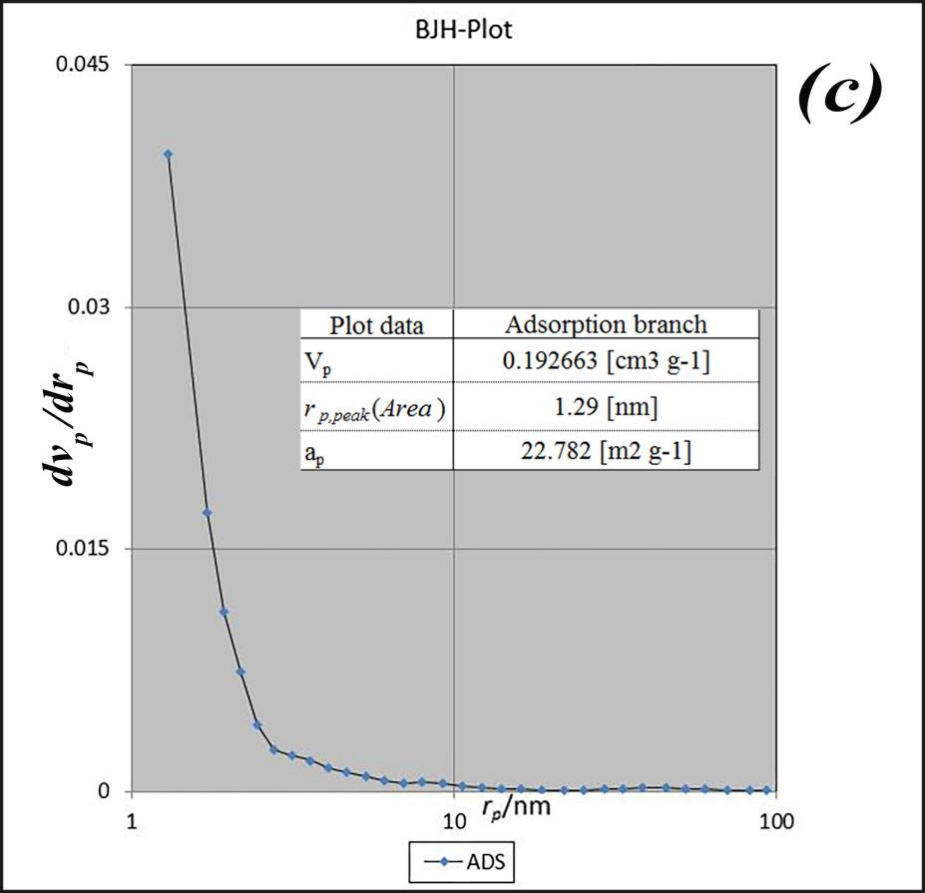
(**a**) Adsorption/desorption isotherms; (**b**) BET and (**c**) BJH plot of nano-MIL-100.

**Figure 8:**
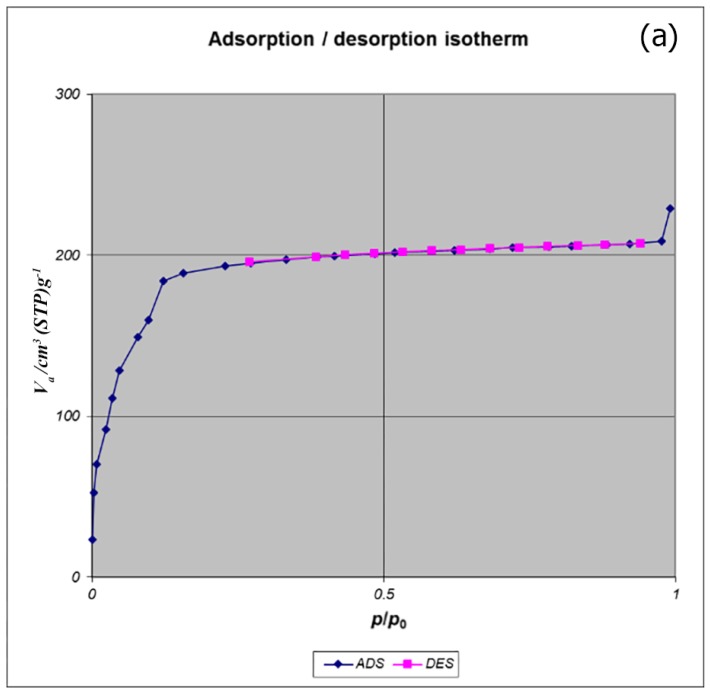

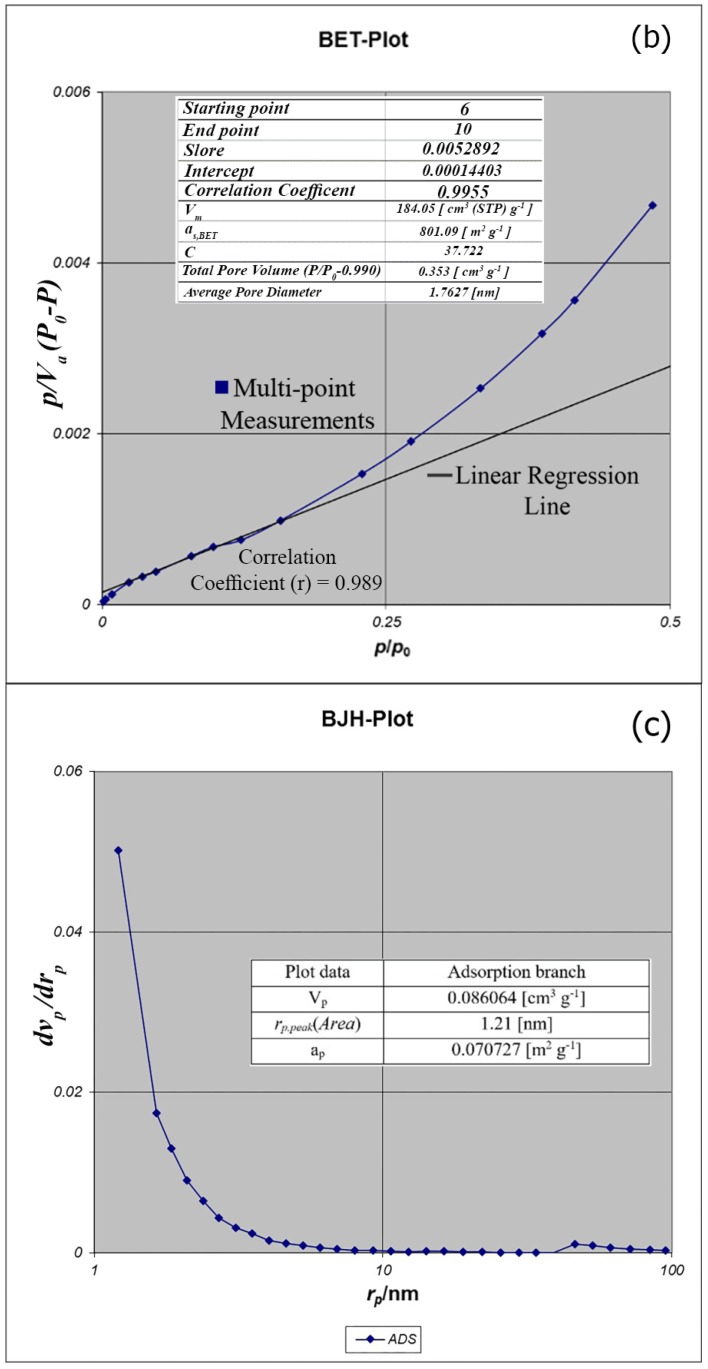
(**a**) Adsorption/desorption isotherms; (**b**) BET and (**c**) BJH plot of TC@MIL-100.

BET analysis for nano-MIL-100 indicates that the available surface area before loading the drug is about 1400 m^2^ g^−1^. BJH analysis revealed that the average pore diameter is 1.29 nm, which demonstrates the micro-porosity of compound. N_2_ sorption/desorption revealed that the pore volume of nano-MIL-100 is 350.07 cm^3^ g^−1^ (Figure 7). The available surface after loading the drug was decreased to 801 m^2^ g^−1^ (Figure 8). The twisting in the last cycle of both TC and DOX makes them suitable for encapsulation in the pores of nano-MIL-100, and BTC rings provide the possibility of forming non-covalent interaction ns between the framework and the drugs. Reduction of the pore volume (to 184.05 cm^3^ g^−1^) and the available surface area is an indication of TC and DOX residing inside the pores of nano-MIL-100(Fe). Considering the fact that both drugs have the same size and behave just like each other during encapsulation in nano-MIL-100, the data of BET analysis for both are the same. Two straight and curved lines could be seen in Figure 7b and Figure 8b which the first one is the linear regression of multi-point measurements. The correlation coefficient, r, of the linear regression is approximately 0.997 and the r^2^ would be 0.995 which would confirm the data of BET analysis. 

**Figure 9:**
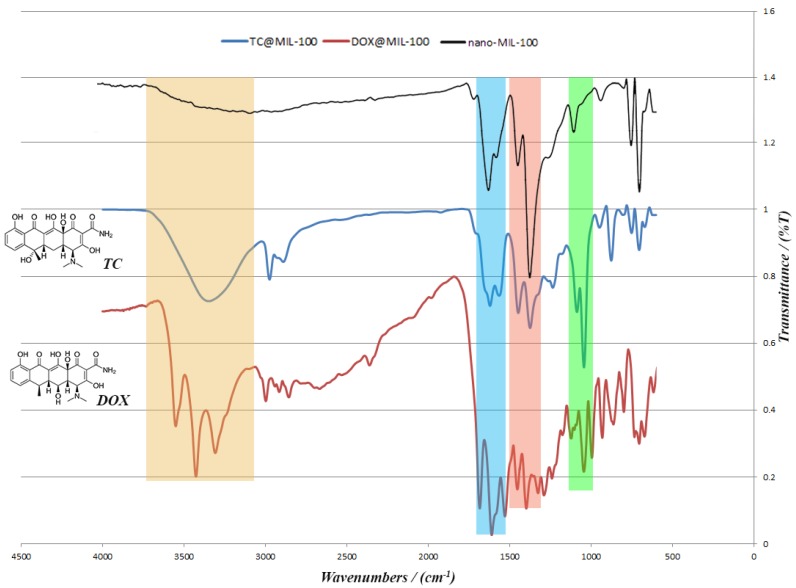
The FT-IR spectra for nano-MIL-100; TC@MIL-100 and DOX@MIL-100.

TC and the DOX were separately loaded into nano-MIL-100 and analyzed by XRD and FT-IR. Both analyses confirmed the loading of drug into the pores of the carrier. It is obvious in Figure 9 that the pattern of FT-IR spectra for DOX@MIL-100 and TC@MIL-100 follow the nano-MIL-100, with some differences. The additional peaks are related to DOX and TC, and it can be concluded that the drugs are well loaded into the carrier. As can be seen in the yellow region, there is a broad peak for TC@MIL-100 at 3346 cm^−1^. This broad peak demonstrates the presence of O–H and N–H. Alongside this peak, there are two peaks in the pink region (1446 and 1373 cm^−1^) that indicate the presence of O–H bending in the plane. The amine peaks are usually weak, and the amount of O–H in this structure shields the peak of the amine group. The presence of amine groups could be further confirmed by the bending vibrations that belong to NH_2_ scissoring in the blue region (1620 and 1563 cm^−1^). Two stretching peaks in the green region (1042 and 1085 cm^−1^) could also be assigned to C–N bonds. These three regions are also crystal clear in the DOX@MIL-100 spectrum, which indicates the presence of same groups with the same effect. The main difference between TC@MIL-100 and DOX@MIL-100 relies on the yellow region, where the peak of TC is broad, and the peak of DOX is separated into three sharp peaks. The possible reason for this could be related to a different position for one hydroxyl group in TC in comparison to DOX, which leads to a different space group. The space group of Doxycycline monohydrate is *P*2_1_, and for Tetracycline hydrochloride is *P*2_1_2_1_2_1_. So two different space groups result in slight differences in FT-IR spectrum.

**Figure 10:**
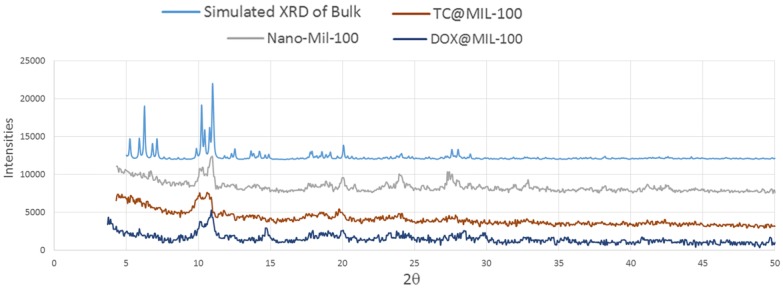
The XRD pattern of Simulated, Nano-MIL-100, TC@MIL-100, and DOX@MIL-100 (from top to bottom).

The XRD pattern of the nano-MIL-100 powder has been compared with the simulated XRD pattern from single crystal X-ray data of MIL-100 as illustrated in Figure 10. Acceptable matches are observed for the patterns, indicating that the nano-sized MIL-100 obtained by the sonochemical/hydrothermal method has an identical structure to that of the crystalline MIL-100 determined by single crystal diffraction. Upon the examination of morphology of the host material before and after drug encapsulation, DOX and TC has not contributed in the pattern of XRD, so it can be concluded that they are loaded into the pores of nano carrier and the structural integrity of MIL-100 (Fe) was retained in hosting DOX and TC.

**Figure 11:**
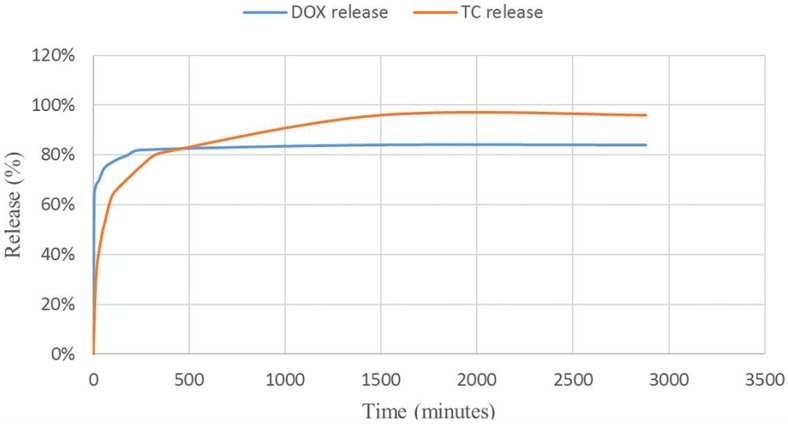
Release curves for TC@MIL-100 and DOX@MIL-100.

The release profiles of TC and DOX were investigated in Simulated Gastric Fluid (SGF) and Phosphate-Buffered Saline (PBS), respectively (see Figure 11). The release curves and calculations showed enhancement in total release of tetracycline and doxycycline compared to the reported methods. Comparing the TC with the DOX revealed that the DOX is released in higher amounts in the first minutes. The interesting point of this research is the mediating effect of MIL-100 on these antibiotics. DOX, as an insoluble drug in water has loaded into the pores of MIL-100 via non-covalent interactions. Considering the fact that DOX is not aggregated and it is well dispersed into the pores of nano-MIL-100, by immersing DOX@MIL-100 in PBS solution, the solvent reaches to the pores and has more time to solve the encapsulated drug, so the frontier molecules in first layers will rapidly dissolve in the PBS solution, and the fast kinetics in the first few minutes can be assigned to this phenomenon. The amazing result of DOX release in PBS reveals that using this platform could increase the amount of released drug 25% more than conventional methods. In contrast, TC, as a very soluble drug in water, shows slower release due to the formation of stronger bonds with the carrier. As the SGF solution reaches the pores, it takes more time to dissolve the TC encapsulated into the pores. It is noteworthy that almost all of the drug dissolves in the solvent, and after 48 h, 96% of the TC releases into SGF. Our findings show a great match with the results of Singco et al. for aspirin release from nano-MIL-100 [17]. The degree of degradation of nano-MIL-100 as the carrier in the acidic medium is slower than an alkaline medium. It could be related to the building blocks of MIL-100. The pKa of trimesic acid is <5.5 and the pH of PBS is 7.4. Therefore, at this pH, trimesic acid was deprotonated, which resulted in its dissociation from the framework and DOX released faster than TC. On the other hand, for MIL-100(Fe) disc immersed in pH 1.2 (HCl), the degradation is gradual due to the acidic nature of the medium. So the TC would be shielded from unnecessary interactions in the stomach as its place of action.

## 3. Materials and Methods

All reagents for the synthesis and analysis were commercially available from Merck and Aldrich Company (Darmstadt, Germany) and used as received. The ultrasonic synthesis was carried out on a SONIC 3MX, (maximum 160 W at 37 kHz) (Manchester, UK). The hydrothermal oven used in this research was MEMMERT UF 55 Plus (Schwabach, Germany). Melting points were measured on an Electrothermal 9100 apparatus (Stone ST15 0SA, UK). UV analyses were conducted by a Perkin Elmer–Lambda 35 UV-Visible spectrometer (Shelton, CT, USA). FT-IR spectra were recorded on Bruker Equinox 55 spectrometer (Bruker Optics, Marne la Vallée, France) equipped with a single reflection Diamond ATR system over the range of 600–4000 cm^−1^. Scanning Electron Microscopy (SEM) was performed on an MIRA3 TESCAN (Brno, Česká republika). The simulated XRD powder pattern based on single crystal data were prepared using Mercury software version 3.8 (Cambridge, UK). X-ray powder diffraction (XRPD) measurements were performed using a Philips PW1730 diffractometer with monochromated Cu-Kα radiation (λ = 1.54056 Å) (Amsterdam, The Netherlands), with the step size of 0.05 and 1s per step. Data analysis and FWHM calculation of XRD peaks were performed by OriginPro 2017 software (OriginLab Corporation, Northampton, MA, USA). The BET analyzer model was a BELSORP-mini II analyzer (Ankersmid Ltd., Nijverdal, Australia). Particle size distribution (DLS), PDI index, and Zeta potential were measured by Zetasizer ZEN3600 (Malvern Instruments Ltd., Worcestershire, UK).

The carrier was synthesized via Horcajada’s et al. method with some modifications [14]. One mmol (0.058 g) of Fe(NO_3_)_3_·9H_2_O and 0.67 mmol (0.1408 g) of BTC (Benzene tricarboxylic acid) were dissolved in 0.6 mmol (0.025 mL) HNO_3_, 2.28 mmol (0.04 mL) HF and 5 mL H_2_O. The solution was placed in the ultrasonic bath for 5 min, transferred to a steel autoclave, sealed, heated for 12 h until reached to 140 ºC, kept at that temperature for 36 h, and then cooled to room temperature in 24 h. The reddish brown sediments were then collected and washed with hot ethanol and deionized water for five or six times until all unreacted ligands washed away.

The process of encapsulating the drug was initiated after preparing the nanocarrier. Tetracycline was dissolved in 2 mL of ethanol. For acquiring a 250 mg tablet of drug@carrier, 0.01 g of tetracycline should be weighed against 0.03 g of the carrier. The nano-MIL-100 powder was then added to the solution of TC and steered for 48 h until all of the drug molecules entrapped in the pores of the carrier. After that, the brown solution transferred to a rotary evaporator until the solution evaporated and sediments of drug@carrier remained. In the case of DOX, 0.0107 g of DOX was dissolved in 12 mL of ethanol, and the same procedure was repeated for DOX.

For the quantification of TC and DOX, loaded in MIL-100(Fe), the ethanolic TC and DOX standards for calibration were separately injected into UV capillaries. Calibration curves were prepared from standard solutions of TC and DOX separately. Samples of 1, 3, 5, 10, 15, 25, 50, 75 and 100 ppm of both drugs were prepared and analyzed by UV-Vis spectroscopy (Figures S1 and S2).

The release profile of TC was investigated in Simulated Gastric Fluid (SGF). The SGF was prepared without pepsin by the Galia et al. method [27]. The temperature of the release medium was set on 37 ℃, and the pH adjusted to 1.2. A 0.015 g sample of TC@MIL-100 was dissolved in SGF, and the whole container was placed in an oil bath. After 5 min, the container was removed from stirrer and abandoned for 2 min. Then, 0.5 mL of upper solution was collected and transferred to a test tube. For recovery, 0.55 mL of SGF was added to the container. This process was repeated at predetermined time intervals (15 min, 30 min, and 45 min, 1 h, 90 min, and 2 h, 3 h, 4 h, 5 h, 6 h, 24 h, and 48 h). The released samples were immediately transferred to UV-Vis spectrometer and prepared for analyses at a wavelength of 270 nm.

The release profile of DOX was monitored in Phosphate Buffer Saline (PBS). The PBS solution was prepared by Dulbecco and Vogot method [28]. The pH was controlled and kept at 7.4. The rest of the process and analyses were carried out similar to the case of TC.

## 4. Conclusions

Two antibiotics with different solubility, tetracycline hydrochloride and doxycycline monohydrate, were loaded separately on the synthesized nano-sized MIL-100(Fe), which was used as the carrier. The crystallinity and purity of MIL-100(Fe) was confirmed using several characterization techniques including XRD, FT-IR, BET and DLS, and remained intact after encapsulation of the drugs. It was revealed that nano-sized MIL-100(Fe) has a mediating effect on these antibiotics. The result of DOX release in PBS reveals that using this platform could increase the amount of released drug to 25% more than that of conventional methods. In contrast, TC, as a highly soluble drug in water, shows slower release due to the formation of stronger bonds with the carrier. The difference in the pH of the media also affected the release of drugs and resulted in a faster release for DOX compared with TC. Interestingly the slower kinetics of TC lead to 96% of the drug being released in acidic medium, which is much higher than previous reports. 

A sustained release and slow kinetics of releasing of antibiotics into the body would be desirable for treatment of infections that need more time to cure. The results of this research could be followed by pharmacists who are studying methods to increase the time intervals of taking antibiotic pills orally, in order to lower the side effects of consuming too much antibiotics.

## Supplementary Materials

The following are available online at www.mdpi.com/2079-4991/7/8/215/s1, Equation ES1: Calculations on XRD pattern of nano-MIL-100, Figure S1: Calibration curve for tetracycline in different concentrations, Figure S2: Calibration curve for Doxycycline in different concentrations, Table S1: Tetracycline structural analysis, Table S2: Doxycycline structural analysis.
