# Supplementary material for: Application of Metal-Organic Framework Nano-MIL-100(Fe) for Sustainable Release of Doxycycline and Tetracycline
Source: Nanomaterials (Basel). 2017 Aug 6;7(8):215. doi: 10.3390/nano7080215 (PMC5575697; doi:10.3390/nano7080215)
Supplement: Supplementary file 1 [file nanomaterials-07-00215-s001.pdf]

Supporting information for

Article

Application of Nano-MIL-100(Fe) for Sustainable Release of Doxycycline and Tetracycline

Seyed Dariush Taherzade [1], Janet Soleimannejad [1,*], and Aliakbar Tarlani [2]

* Correspondence: janet_soleimannejad@khayam.ut.ac.ir; Tel.: +98-21- 66495291

Table S1. Tetracycline structural analysis.

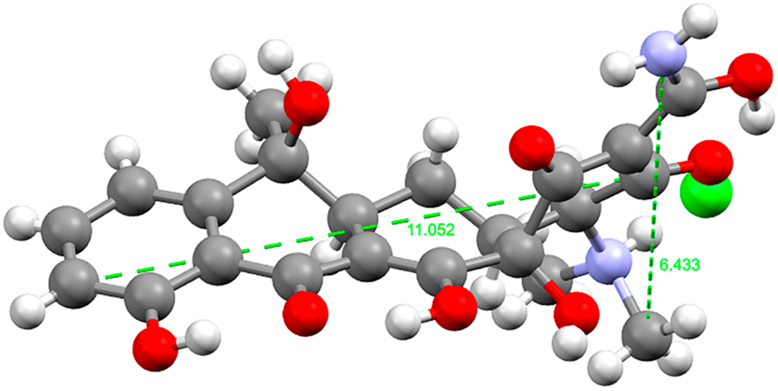

Chemical name	Tetracycline Hydrochloride
Molecular Formula	$C_{22}H_{24}N_2O_8$
Molecular Weight	444.44 g/mol
IUPAC name	(4S,4aS,5aS,6S,12aR)-4-(dimethylamino)-1,6,10,11,12a-pentahydroxy-6-methyl-3,12-dioxo-4,4a,5,5a-tetrahydrotetracene-2-carboxamide
Hydrogen Bond Donor Count	6
Hydrogen Bond Acceptor Count	9
Rotatable Bond Count	2

Table S2. Doxycycline structural analysis.

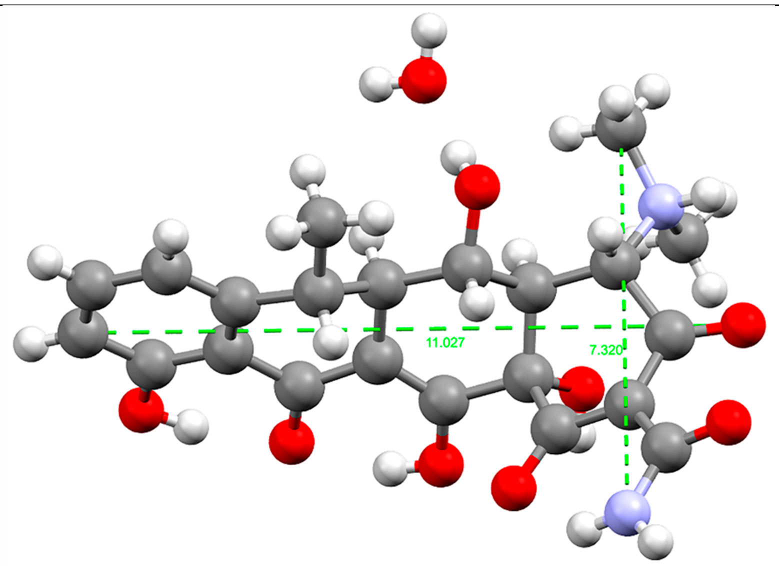

Chemical name	Doxycycline Monohydrate
Molecular Formula	$C_{22}H_{26}N_2O_9$
Molecular Weight	462.455 g/mol
IUPAC name	(4S,4aR,5S,5aR,6R,12aR)-4-(dimethylamino)-1,5,10,11,12a-pentahydroxy-6-methyl-3,12-dioxo-4a,5,5a,6-tetrahydro-4H-tetracene-2-carboxamide;hydrate
Hydrogen Bond Donor Count	7
Hydrogen Bond Acceptor Count	10
Rotatable Bond Count	2

*The above information is extracted from CIF (Crystal Information File).

The Scherrer Equation, $\boldsymbol{\tau} = \frac{\boldsymbol{K \times \lambda}}{\boldsymbol{B \times Cos\theta}}$, as an accepted technique to calculate the nano-crystallite sizes (τ) by XRD, relates the diffraction from brag planes (2θ) to the wavelength of X-ray radiation source (λ) and the full width at half maximum of peaks (B) which is measured by applying the Gaussian profile on the main peak.

ES 1. Calculations on XRD pattern of nano-MIL-100.

$\boldsymbol{\lambda = 1.54}$ Å , $\boldsymbol{K = 0.89}$,

$B = FWHM = 0.0016,$

$$\tau = \frac{K \times \lambda}{B \times Cos\theta} = \frac{0.89 \times 0.154}{0.0016 \times 0.97} = 88\ nm,$$

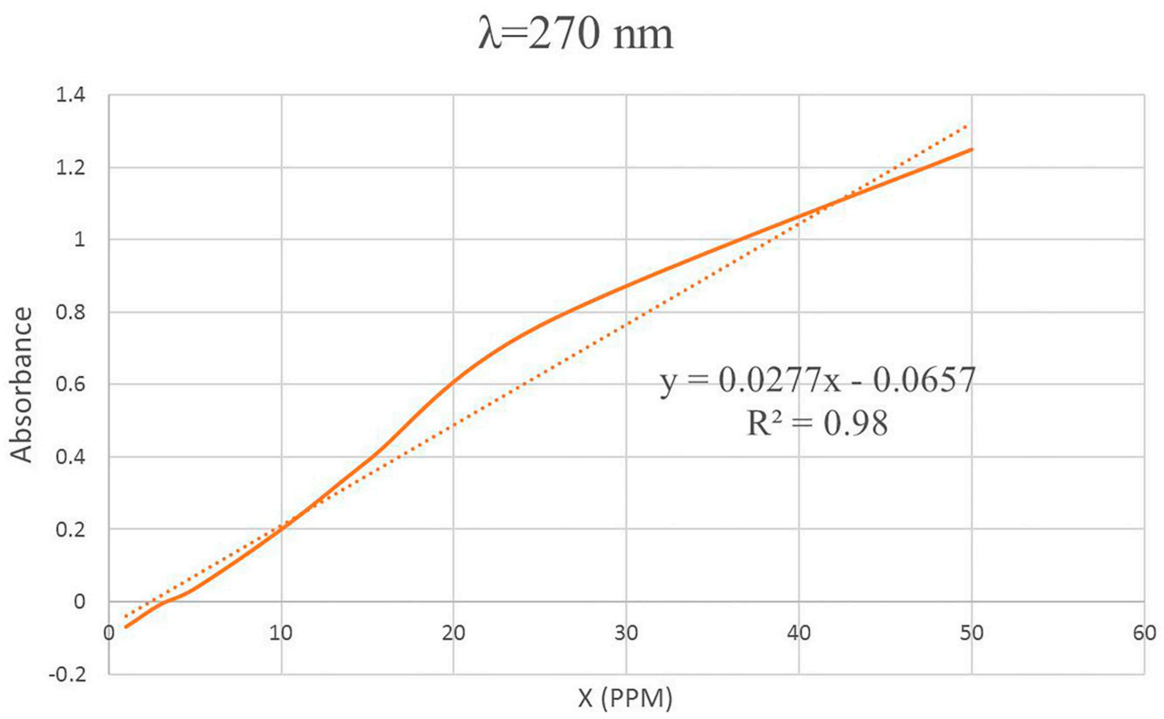

Figure S1. Calibration curve for tetracycline in different concentrations.

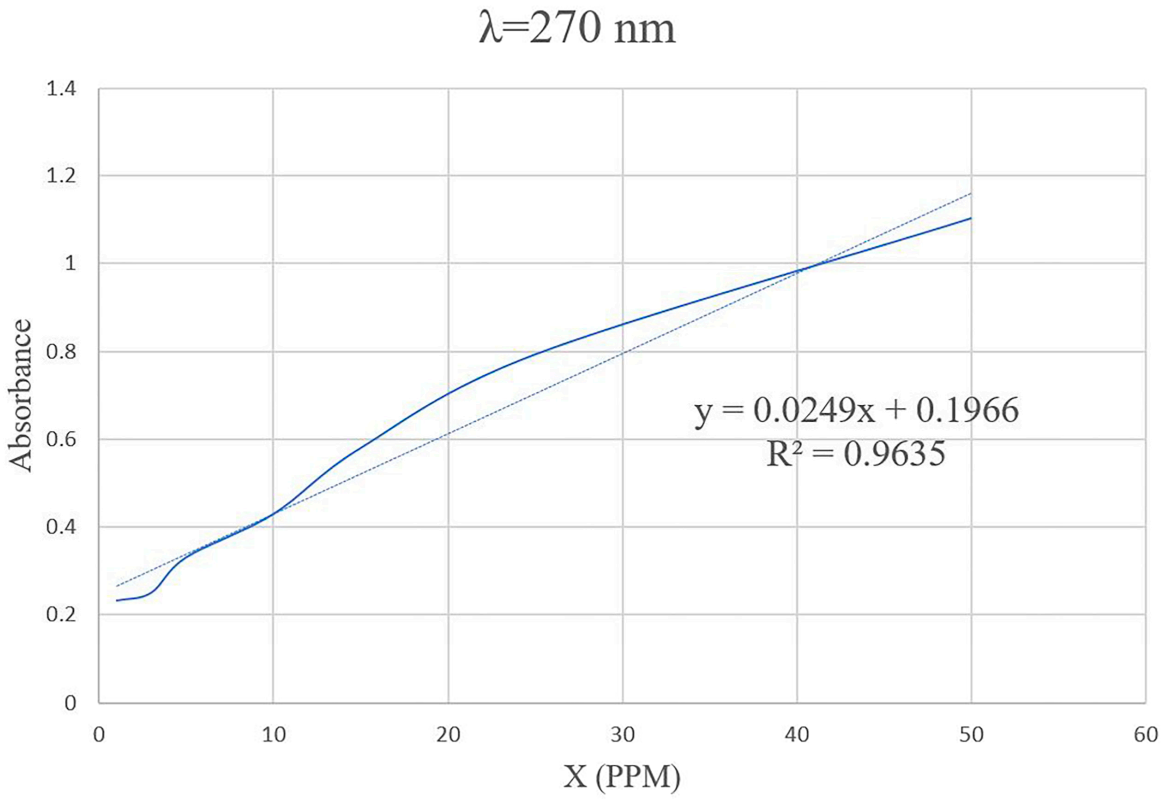

Figure S2. Calibration curve for Doxycycline in different concentrations.
